# Health-related quality of life for pre-diabetic states and type 2 diabetes mellitus: a cross-sectional study in Västerbotten Sweden

**DOI:** 10.1186/s12955-014-0150-z

**Published:** 2014-10-24

**Authors:** Anne Neumann, Olaf Schoffer, Fredrik Norström, Margareta Norberg, Stefanie J Klug, Lars Lindholm

**Affiliations:** Epidemiology and Global Health, Department of Public Health and Clinical Medicine, Umeå University, SE-901 87, Umeå, Sweden; Cancer Epidemiology, University Cancer Center, University Hospital, Technische Universität Dresden, Fetscherstr. 74, 01307 Dresden, Germany

**Keywords:** Health utility, Normal glucose tolerance, Impaired fasting glucose, Impaired glucose tolerance, Type 2 diabetes mellitus, Sweden, SF-36, SF-6D, Health-related quality of life, Beta regression

## Abstract

**Background:**

Type 2 diabetes (T2D) decreases health-related quality of life, but there is a lack of information about the health status of people in pre-diabetic states. However, information on health utility weights (HUWs) for pre-diabetic states and T2D are essential to estimate the effect of prevention initiatives. We estimated and compared HUWs for healthy individuals, those with pre-diabetes and those with T2D in a Swedish population and evaluated the influence of age, sex, education and body mass index on HUWs.

**Methods:**

Participants of the Västerbotten Intervention Program, Sweden, between 2002 and 2012, who underwent an oral glucose tolerance test or indicated they had T2D and who filled in the Short Form-36 questionnaire (SF-36) were included. Individuals were categorized as healthy, being in any of three different pre-diabetic states, or as T2D. The pre-diabetic states are impaired fasting glucose (IFG), impaired glucose tolerance (IGT) or a combination of both (IFG&IGT). The SF-6D index was used to convert SF-36 responses to HUWs. HUWs were stratified by age, sex, education and body mass index. Beta regression analyses were conducted to estimate the effect of multiple risk factors on the HUWs.

**Results:**

In total, 55 882 individuals were included in the analysis. The overall mean HUW was 0.764. The mean HUW of healthy individuals was 0.768, 0.759 for those with IFG, 0.746 for those with IGT, 0.745 for those with IFG&IGT, and 0.738 for those with T2D. In the overall model, all variables except underweight vs. normal weight were significantly associated with HUW. Younger age, male sex, and higher education were associated with increased HUW. Normal weight, or being overweight was associated with elevated HUW, while obesity was associated with lower HUW.

**Conclusions:**

Healthy individuals had higher HUWs than participants with T2D, while individuals with IFG, IGT or IFG&IGT had HUWs that ranged between those for NGT and T2D. Therefore, preventing the development of pre-diabetic states would improve health-related quality of life in addition to lowering the risk of developing T2D.

## Background

Type 2 diabetes mellitus (T2D) is a severe disease with an estimated number of cases exceeding 370 million globally and 390 000 in Sweden in 2012 [1]. These numbers are expected to increase to over 550 million and 432 000 by 2030, respectively [1]. Several studies have shown that prevention of T2D is possible by changing behavior, such as through physical activity and diet [2-4]. However, prevention does not start with the onset of the disease, but needs to address the early stages of T2D. Therefore, the best scenario involves preventing the development of pre-diabetic states among healthy individuals.

T2D and pre-diabetic states can be detected using a standardized oral glucose tolerance test and medical classifications, such as those of the World Health Organization [5]. According to this classification of blood glucose levels, a person can be categorized as normal glucose tolerant (NGT), i.e. healthy, being in a pre-diabetic state, or as having T2D. The pre-diabetic states are impaired fasting glucose (IFG), impaired glucose tolerance (IGT), and a combination of both (IFG&IGT). Subjects in any of these three pre-diabetic states have moderate to severe insulin resistance, impaired insulin secretion and/or insulin sensitivity, and each state has distinct pathophysiologic etiologies and risks of developing into T2D [6,7].

Disease, such as T2D, is assumed to reduce the health-related quality of life (HRQoL) of those affected. The estimation of HRQoL to assess either health status or the benefit of an intervention is a cornerstone in health economic evaluation. Quality-adjusted life years (QALYs) quantify HRQoL and combine the length of life and the preference weight for a particular health state into a single measure [8]. The intensity in the preference weight is measured as a “health utility weight” (HUW). A HUW of 1.0 indicates “perfect health” while a HUW of 0.0 represents being dead.

Models have been developed to estimate the cost-effectiveness of diabetes prevention initiatives which focus on lifestyle change by calculating the cost per QALY gained [9]. However, the validity of such models can be challenged, as not all necessary data are available. One important gap is information about HUWs from the same population to allow comparability between all health states in such models [9]. No prior research has extracted HUW from NGT, IFG and/or IGT and T2D from the same source population in a Swedish population. A recent study in Finland is, according to our knowledge, the only investigation that examined HUWs for NGT, IFG, IGT and T2D [10].

In this study, we aim to estimate and compare HUWs for individuals with NGT, IFG, IGT and T2D in a Swedish population, and to evaluate the influence on HUWs of age, sex, education and body mass index (BMI), factors known to independently influence HRQoL.

## Methods

### Study population

Participants of the Västerbotten Intervention Program (VIP) who underwent an oral glucose tolerance test or indicated that they had T2D and who filled in the Short Form-36 questionnaire (SF-36) were included in this cross-sectional study. As the SF-36 questionnaire was not included in the VIP before 2003, inclusion in the study was limited to individuals with examination dates between January 2003 and February 2012. The VIP was initiated in 1985 with the aim of reducing morbidity and mortality from cardiovascular disease and diabetes [11]. All people at ages 40, 50 and 60 living in the Swedish county of Västerbotten are eligible for inclusion in the VIP, and are invited to screening and health counseling conducted by their primary care provider [11]. Thirty-year olds were also invited until 1996. Part of this screening is an oral glucose tolerance test with a 75 g oral glucose load conducted according to standards of the World Health Organization [5]. Based on this test, VIP participants were categorized into one of the following glucose groups NGT, IFG, IGT, IFG&IGT, or T2D according to the 1999 WHO classification [5]. Further, height, weight, blood pressure and plasma lipids are measured, and each VIP participant is asked to complete a set of questionnaires, including questions about physical activity, tobacco use and dietary habits. It has been shown that the participants of VIP are representative of the county of Västerbotten [12]. The VIP is described in more detail elsewhere [11].

### Health utility weights

The SF-36 is a standardized generic questionnaire comprising 36 questions designed to assess self-perceived health status. It is a psychometric measure that produces a profile of eight dimensions [13]. The scoring of the SF-36 is not preference-based and assumes that the items are of equal importance [14]. The SF-36 has been reported as valid and reliable in healthy populations and diabetes patients [15-18]. However, for the estimation of HUW, preference-based estimates are necessary. Therefore, the SF-36 must be converted to preference-based items for the development of the HUW. The Short Form-6D (SF-6D) questionnaire was developed to obtain HUWs from the SF-36 questionnaire for use in health economic evaluations and links between psychometric and preference/utility-based measures [19]. A subset of 11 questions from the SF-36 is included in the SF-6D and weighted according to Brazier and Roberts (2004) [14]. The eight dimensions of the SF-36 were reduced to six SF-6D dimensions: physical functioning, role limitations, social function, bodily pain, mental health, and vitality [19]. No limitation in any of the dimensions means no subtraction from the baseline value of 1.0, i.e. perfect health. The higher the limitation in each domain, the higher the subtraction from the baseline [14]. The summation of the six dimensions constitutes the HUW. The SF-36 and its conversion to SF-6D for HUWs are widely used in health economic and epidemiological studies [19]. The SF-6D valuation was shown to be representative for the population of the United Kingdom [20].

The responses of the SF-36 questionnaires were converted into HUWs using the SF-6D index. For conversion, we used the SAS code “Sf6d_sf36v1_UK_mod.sas”, obtained from the University of Sheffield [14]. The SF-36 version 1.0 UK was used in the Swedish language in the VIP for this analysis. Mean HUW scores were estimated. However, due to the expected asymmetric distribution of the HUWs in the study population, median HUW scores were also calculated.

### Risk factors

The HUWs were stratified by four potential risk factors (age, sex, education and BMI), which are known to independently influence HUWs and which are easy to measure. Box plots were used to illustrate stratified HUWs. Outliers were not displayed, as they would distort the boxplots. Education was classified as basic (only compulsory school or <10 years of formal education), middle (10–12 years of formal education) or high (university or ≥13 years of formal education). BMI was classified as underweight (<18.5), normal weight (18.5-24.9), overweight (25.0-29.9) or obese (≥30.0).

### Statistical analyses

Chi-square tests were conducted to test the significance of the count data of the description of the study population (Table 1). The differences of the mean HUWs between the glucose groups were tested using the Kruskal-Wallis test. To identify for which glucose groups there were differences, the post-hoc Mann–Whitney tests with the Bonferroni-Holm procedure to adjust for multiple comparisons was used [21].

**Table 1:** **Study population, total, by age, sex, education and body mass index** ^**1**^

	**Healthy**		**Pre-Diabetes**						**Diabetes**			
**Characteristics**	**NGT**		**IFG**		**IGT**		**IFG & IGT**		**T2D**		**Total**	
	**n**	***%***	**n**	***%***	**n**	***%***	**n**	***%***	**n**	***%***	**n**	***%***
Total number	43 586	*100.0*	5 629	*100.0*	2 440	*100.0*	1 232	*100.0*	2 995	*100.0*	55 882	*100.0*
**Age in years**												
40	15 652	*35.9*	1 339	*23.8*	418	*17.1*	168	*13.6*	350	*11.7*	17 927	*32.1*
50	14 707	*33.7*	1 846	*32.8*	706	*28.9*	358	*29.1*	798	*26.6*	18 415	*33.0*
60	13 227	*30.4*	2 444	*43.4*	1 316	*53.9*	706	*57.3*	1 847	*61.7*	19 540	*35.0*
**Sex**												
Male	21 076	*48.4*	2 973	*52.8*	1 050	*43.0*	638	*51.8*	1 844	*61.6*	27 581	*49.4*
Female	22 510	*51.7*	2 656	*47.2*	1 390	*57.0*	594	*48.2*	1 151	*38.4*	28 301	*50.6*
**Education**												
Basic^2^	5 655	*13.0*	1 052	*18.7*	508	*20.8*	279	*22.7*	753	*25.1*	8 247	*14.8*
Middle^3^	22 606	*51.9*	2 938	*52.2*	1 275	*52.3*	654	*53.1*	1 568	*52.4*	29 041	*52.0*
High^4^	15 053	*34.5*	1 596	*28.4*	634	*26.0*	286	*23.2*	639	*21.3*	18 208	*32.6*
*Missing*	*272*	*0.6*	*43*	*0.8*	*23*	*0.9*	*13*	*1.1*	*35*	*1.2*	*386*	*0.7*
**Body mass index, BMI** ^5^												
Underweight, <18.5	524	*1.2*	45	*0.8*	31	*1.3*	1	*0.1*	14	*0.5*	615	*1.1*
Normal, 18.5 - 24.9	18 917	*43.4*	1 735	*30.8*	688	*28.2*	221	*17.9*	462	*15.4*	22 023	*39.4*
Overweight, 25.0 – 29.9	17 669	*40.5*	2 420	*43.0*	1 070	*43.9*	521	*42.3*	1 173	*39.2*	22 853	*40.9*
Obesity, ≥30.0	6 293	*14.4*	1 403	*24.9*	637	*26.1*	486	*39.5*	1 320	*44.1*	10 139	*18.1*
*Missing*	*183*	*0.5*	*26*	*0.5*	*14*	*0.6*	*3*	*0.2*	*26*	*0.9*	*252*	*0.5*

^1^Chi-square test was used to test for dependencies between glucose tolerance groups and age, sex, education level and body mass index respectively. All comparisons were significant (p < 0.001).

^2^“compulsory school” or “less than 10 years of education in school”.

^3^“10-12 years of education in school”.

^4^“university” or “education of more than 12 years in school”.

^5^BMI = [weight in kg]/[height in m]^2^.

Beta regression as introduced by Ferrari and Cribari-Neto [22] was used to estimate the effect of multiple risk factors on the HUW, as the distribution of the HUWs fitted well with a beta distribution [23]. Beta regression can be expressed as $g\left({\mu}_t\right)={\displaystyle {\sum}_{i=1}^k{x}_{ti}{\beta}_i}$ which follows the beta distribution with mean (*E*(*y*) = *μ*) and variance $\left(var(y)=\frac{\mu \left(1-\mu \right)}{1+\phi}\right)$ where g(^.^) is the logit link function, *x*_*ti*_ represents the *t*-th observation and *β*_*i*_ the unknown regression parameter of the *i*-th covariate [22]. Beta regression has been applied to analyze HRQoL [24-27]. It presents the most flexible way to simultaneously estimate the structure of dependence and the distribution parameters, as the dependent variable is beta distributed. As covariates were present, the alternative parameterization with location parameter and scale parameter was used [27]. This is important for the interpretation of the regression parameters and thus the model equation. The mean HUW is represented by *μ* (alternative parameterization). In the conventional parameterization, the shape parameters need to be transformed for an equivalent statement.

The significance of the beta regression models was tested with the Wald test. The McFadden’s pseudo-R^2^ was estimated as goodness-of-fit-criterion using the following formula: McFadden’s pseudo-R^2^ = 1 – ln L_null_/ ln L_fit_, where L_null_ is the log-likelihood of the null model, and L_fit_ is the log-likelihood of the fitted model [27].

The variable age was used as a continuous variable, even though only three ages, i.e. 40, 50, 60, were possible. As the variables education and BMI were ordinal and not interval scaled, dummy variables were created to evaluate the differences between basic vs. middle and basic vs. high education as well as normal weight vs. underweight, normal weight vs. overweight and normal weight vs. obese. Basic education and normal weight were used as reference categories.

Conversion from SF-36 to SF-6D was conducted with SAS 9.22. For all other statistical analyses STATA/SE 11.0 was used. The significance level for all statistical tests was 0.05.

Ethical approval for this study was received from the Regional Ethics Board Dnr 08-131 M at Umeå University, Sweden. All subjects gave informed consent to future research before their VIP-examination.

## Results

### Study population

The total number of individuals in our analysis was 55 882 (Table 1). The age distribution of study participants was relatively equally distributed among 40, 50 and 60 year olds (32.1, 33.0 and 35.0%, respectively), with a mean age of 49, 52, 54, 54 and 55 years among individuals with NGT, IFG, IGT, IFG&IGT and T2D, respectively. Approximately half (49.4%) of the study population was male. Most study participants had middle education (52.0%), followed by high (32.6%) and basic (14.8%). The majority of the participants was either overweight (40.9%) or had normal weight (39.4%) while 18.1% were obese and 1.1% was underweight (Table 1). In total, the majority was categorized as NGT (n = 43 586, 78.0%), while 5 629 individuals had IFG (10.1%), 2 440 IGT (4.4%), 1 232 IFG&IGT (2.2%) and 2 995 T2D (5.4%).

People in the older age group tended to have more severe glucose group (NGT: 30.4% vs. T2D: 61.7% among 60-year olds compared to NGT: 35.9% vs. T2D: 11.7% among 40-year olds). Further, participants with T2D had higher BMI (83.3% obese or overweight) comparing participants with NGT (54.9% obese or overweight) (Table 1). All comparisons for dependencies between glucose tolerance groups and age, sex, education level and body mass index respectively were significant (p < 0.001).

### Health utility weights

The total number of individuals with estimated HUW was 52 606. There were 3 276 individuals (5.9%) in the data set which could not contribute to the HUW calculations (Table 2) due to missing values in the answers of the SF-36. The overall mean HUW was 0.764. The mean HUW of healthy individuals was 0.768, 0.759 for those with IFG, 0.746 for those with IGT, 0.745 for those with IFG&IGT, and 0.738 for those with T2D. The HUWs depend on the glucose groups (p <0.001). Multiple pairwise comparisons indicated differences for all glucose groups besides comparing IGT with T2D, IFG&IGT with T2D and IGT with IFG&IGT.

**Table 2:** **SF-6D domains and health utility weights**

**SF-6D domains**						
**Mean (SD)** ^**1**^	**NGT**	**IFG**	**IGT**	**IFG&IGT**	**T2D**	**Total**
n	41 208	5 275	2 261	1 122	2 740	5 2606
Physical functioning	1.840 (1.12)	2.013 (1.23)	2.245 (1.32)	2.276 (1.34)	2.393 (1.40)	1.914 (1.18)
Role limitations	1.535 (1.26)	1.621 (1.41)	1.747 (1.54)	1.825 (1.69)	1.830 (1.58)	1.574 (1.32)
Social function	2.446 (1.03)	2.491 (1.07)	2.602 (1.23)	2.568 (1.16)	2.606 (1.24)	2.468 (1.06)
Bodily pain	2.416 (1.44)	2.503 (1.46)	2.668 (1.50)	2.726 (1.52)	2.758 (1.59)	2.460 (1.46)
Mental health	1.753 (1.08)	1.767 (1.09)	1.807 (1.14)	1.836 (1.22)	1.870 (1.27)	1.770 (1.10)
Vitality	2.666 (1.19)	2.713 (1.25)	2.789 (1.28)	2.800 (1.34)	2.860 (1.40)	2.689 (1.22)
**Health utility weight** ^**2**^						
Mean (SD)^1^	0.768 (0.10)	0.759 (0.11)	0.746 (0.11)	0.745 (0.11)	0.738 (0.12)	0.764 (0.10)
Median	0.793	0.788	0.772	0.772	0.765	0.789
1^st^-3^rd^ quartile	0.713-0.830	0.681-0.830	0.669-0.830	0.667-0.830	0.639-0.830	0.700-0.830
Min-Max^3^	0.301-0.943	0.334-0.943	0.301-0.943	0.383-0.943	0.381-0.943	0.301-0.943

^1^SD = standard deviation.

^2^Kruskal-Wallis equality-of-populations rank test, p < 0.001.

^3^Min-Max = range from minimum to maximum value.

### Univariate analysis

**Figure 1:**
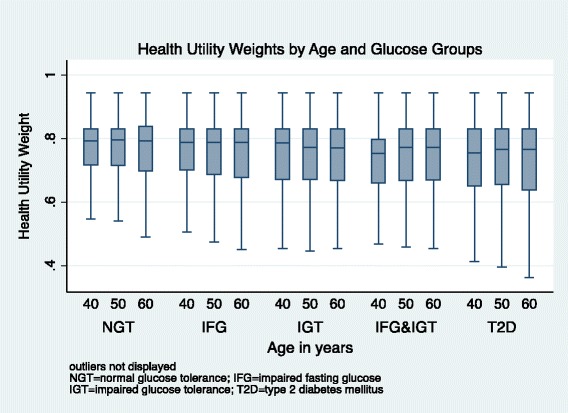
**Health utility weights by age and glucose groups.**

**Figure 2:**
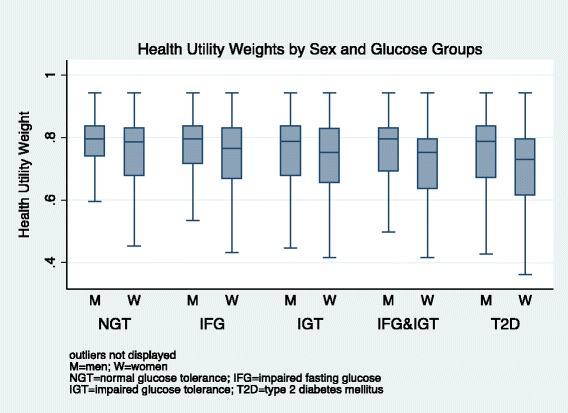
**Health utility weights by sex and glucose groups.**

**Figure 3:**
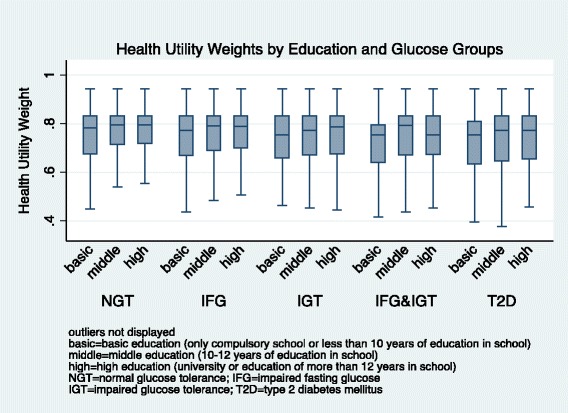
**Health utility weights by education and glucose groups.**

**Figure 4:**
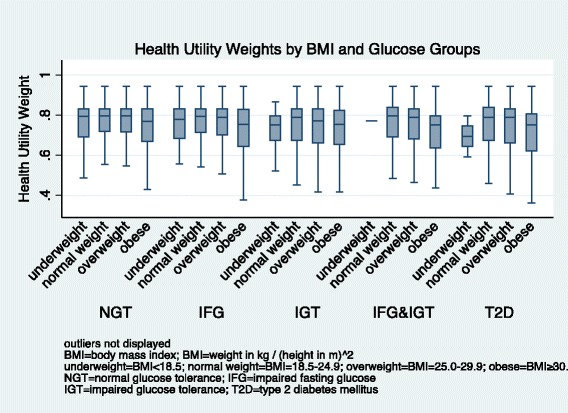
**Health utility weights by BMI and glucose groups.**

In all age groups, we observed the highest median HUW among individuals with NGT, followed by HUWs among pre-diabetic individuals, and lowest HUWs among individuals with T2D (Figure 1). Older age was associated with lower HUW. Women had lower median HUW than men (Figure 2). We observed a decreasing HUW with decreasing glucose group, with highest HUW among individuals with NGT, followed by HUW among individuals with pre-diabetic states, and lowest among individuals with T2D. Higher level of education was associated with higher HUW (Figure 3). BMI was associated with HUW, such that the higher the BMI, the lower the HUW (Figure 4). Underweight individuals had almost equivalent HUW as obese individuals. Underweight individuals with T2D reported lower HUW than obese individuals with T2D.

### Multivariate analysis

52 129 individuals were included in the multivariate regression analyses. Among them, 40 857 had NGT, 5 225 had IFG, 2 236 had IGT, 1 113 had IFG&IGT and 2 698 had T2D. The results of the beta regression showed that all significant factors displayed the same direction, either positive or negative, regardless of glucose group, except for age in the IFG&IGT model (Table 3). However, the McFadden’s pseudo-R^2^ was low for all models, ranging from 0.0120 to 0.0349 (Table 3).

**Table 3:** **Regression coefficients for health utility weight by age, sex, education, BMI and glucose group, beta regression** ^**1**^

**Coefficient (95% CI)** ^**2**^	**NGT**	**IFG**	**IGT**	**IFG&IGT**	**T2D**	**Total**
**Age**	−0.0002	−0.0011	−0.0013	**0.0050**	0.0002	**−0.0008**
(metric)	(−0.0008 - 0.0005)	(−0.0031 – 0.0008)	(−0.0044 – 0.0018)	**(0.0003 – 0.0095)**	(−0.0030 – 0.0035)	**(−0.0014 – -0.0003)**
**Sex**	**−0.1940**	**−0.1855**	**−0.1259**	**−0.1905**	**−0.1968**	**−0.1889**
(reference: male)	**(−0.2048 - -0.1833)**	**(−0.2160 - -0.1549)**	**(−0.1733 - -0.0784)**	**(−0.2558 – -0.1253)**	**(−0.2418 - -0.1519)**	**(−0.1984 - -0.1793)**
**Education**						
(reference: basic)^3^						
Middle^4^	**0.0531**	**0.0571**	0.0175	**0.1304**	0.0489	**0.0552**
	**(0.0364 – 0.0697)**	**(0.0158 – 0.0984)**	(−0.0440 – 0.0791)	**(0.0470 – 0.2137)**	(−0.0050 – 0.1029)	**(0.0411 – 0.0694)**
High^5^	**0.0800**	**0.0645**	0.0595	0.0942	**0.0675**	**0.0813**
	**(0.0624 – 0.0975)**	**(0.0186 – 0.1104)**	(−0.0098 – 0.1288)	(−0.0018 – 0.1901)	**(0.0027 – 0.1322)**	**(0.0661 – 0.0964)**
**Body Mass Index, BMI** ^6^						
(reference: normal, 18.5 - 24.9)						
Underweight, <18.5	0.0033	0.0311	0.2074	0.0803	0.2749	0.0202
	(−0.0448 – 0.0515)	(−0.1404 – 0.2026)	(−0.0095 – 0.4244)	(−1.0080 – 1.1686)	(−0.0711 – 0.6210)	(−0.0250 – 0.0654)
Overweight, 25.0 – 29.9	**0.0549**	**0.0570**	**0.0593**	0.0756	0.0359	0.0590
	**(0.0432 – 0.0665)**	**(0.0212 – 0.0927)**	**(0.0027 – 0.1159)**	(−0.0170 – 0.1682)	(−0.0306 – 0.1023)	(−0.0483 – 0.0696)
Obesity, ≥30.0	**−0.1783**	**−0.1892**	**−0.1288**	−0.1858	**−0.1721**	**−0.1929**
	**(−0.1939 – -0.1626)**	**(−0.2291 – -0.1492)**	**(−0.1920 – -0.0656)**	(−0.2781 – 0.0935)	**(−0.2368 – -0.1075)**	**(−0.2061 – -0.1798)**
**Constant**	**1.5568**	**1.5158**	**0.9803**	0.9812	**0.9028**	**1.5438**
	**(1.4493 – 1.6642)**	**(1.1467 – 1.8849)**	**(0.5003 – 1.4604)**	(−1.2147 – 3.1771)	**(0.1722 – 1.6333)**	**(1.4441 - 1.6435)**
**n**	40 857	5 225	2 236	1 113	2 698	52 129
**McFadden’s pseudo-R** ^**2**^	0.0219	0.0253	0.0120	0.0349	0.0297	0.0238

Significant coefficients are in bold print with significance at a 5% level.

^1^Wald chi2: all five models are significant, p <0.001.

^2^CI = confidence interval.

^3^“compulsory school” or “less than 10 years of education in school”.

^4^“10-12 years of education in school”.

^5^“university” or “education of more than 12 years in school”.

^6^BMI = [weight in kg]/[height in m]^2^.

Using the results of the multivariate regression (Table 3), the mean HUWs (Table 2) can be adjusted for specific sex, age, education and BMI values. For example, the HUW for a women with NGT, aged 40 years with “middle education” and classified as obese would be estimated as the following: 1.5568 (constant) - 0.0002 * 40 (age 40) - 0.1940 (female sex) +0.0531 (middle education) - 0.1783 (obese) =1.2296. Using the inverse logit function on the predicted value 1.2296, one gets exp(1.2296)/(1+ exp(1.2296)) =0.7737. This result is very close to the observed mean value for individuals with NGT (0.768).

In the overall model, all but underweight vs. normal weight were significant factors. Being overweight in comparison to normal weight was associated with increased, whereas being obese was associated with decreased, HUWs. Younger age, male sex, and higher education were associated with higher HUW.

The models including those with NGT or IFG only showed the same pattern as the overall model, except age, which was not significant in both models. For individuals with IGT, age and education were not significant. The model for individuals with IFG&IGT only estimated that an increasing age lead to increasing HUWs. High vs. basic education and overweight vs. normal weight were not significantly associated with HUW. Among individuals with T2D, age, middle vs. basic education, and overweight vs. normal weight were not significantly associated with HUW. All other factors were comparable to the overall model.

## Discussion

### Principal findings

We found that participants with NGT had higher HUWs than those with T2D, while those with IFG, IGT or a combination of both had HUWs that ranged between those for NGT and T2D. All risk factors investigated were associated with HUW, except underweight vs. normal weight. Younger age, male sex, and higher education were associated with increased HUW. Normal weight, or being overweight was associated with elevated HUW, while obesity was associated with lower HUW. However, the McFadden’s pseudo-R^2^ indicated that the model does not explain much variance. It is recognized by Smithson and Verkuilen that the McFadden’s pseudo-R^2^ is often disappointingly small and may not attain the theoretical maximum value of one [27]. The McFadden’s pseudo-R^2^ is not to be interpreted as conventional R^2^ in multiple linear regressions and different definitions of pseudo R^2^ can arrive at very different values.

### Limitations and strengths

Even though the SF-36 – SF-6D converter has only been validated for the UK population, it was used here to draw conclusions about the Swedish population. SF-36 and SF-6D have been widely adopted in Sweden. Still, the responses to the instruments have not yet been translated to HUWs for the Swedish population [28]. Any such bias arising from this would apply to all states and would not influence comparability between states. Therefore, we judged this converter as appropriate for describing HUW in this Swedish population. In addition, our dataset only includes individuals with 40, 50 or 60 years of age. We have, however, used age as continuous instead of categorical variable as we aimed to estimate the effect for all ages in order to make the results applicable to other settings as well. The extrapolation of age far from 40 to 60 years is not recommended, as the variation of estimates will increase according to the distance from the considered age range 40–60 years. In addition, our dataset could not distinguish between T2D cases that were categorized by the oral glucose tolerance test of VIP compared to those who indicated that they had T2D at the time of VIP participation.

The VIP is a population-based longitudinal program from which valid information can be drawn about the whole population of the county of Västerbotten. Every eligible man and woman was invited to the program, thereby minimizing potential selection bias. While no additional attempt was made to encourage populations that are hard to reach, no major differences were found between participants and non-participants in the VIP [12]. One important strength of this study was the large number of participants included in the analyses.

### Other studies

#### HUWs measured with SF-6D

The mean overall HUW was 0.764, which is comparable with results from other studies: the mean baseline SF-6D HUW of participants in the Diabetes Prevention Program in the United States was 0.800 [29], while the HUW calculated for a general Greek population was 0.759 [30]. A study among middle-aged and older Finns revealed very similar mean health utility values among individuals with NGT (0.777), IFG (0.771), IGT (0.759) and newly diagnosed T2D (0.742) [10]. Our estimated HUW for T2D was 0.738 ranging well in the results of other studies [10,31,32].

#### Risk factors

Many studies have also found that older age is associated with lower HUW [30,33,34]. Sex was the only factor that was significant in all regression models leading to the conclusion that women show lower HUWs than men. This has been widely reported in the literature [19,30,33-35]. We found that education was a significant predictor of HUW in some of the models, with lower education being associated with lower HUW, a finding that has been reported elsewhere [30,33]. Our results confirm those of previous studies which reported that obesity is associated with lower HUW [30,33,36]. However, our results contradict with studies indicating that being underweight [36,37] or overweight decreased HUW [19] compared to normal weight. In our study, individuals with underweight and overweight had higher HUW compared to individuals with normal weight.

### Implications for policy makers

We used standardized cost-effectiveness measures for modeling T2D disease interventions, which allow comparison with other published findings and HRQoL of other diseases [38]. Our results allow conclusions to be made about the value of preference for a certain state versus another state enabling cost-effectiveness and cost-utility estimations using cost per QALY gained. Our findings show that early prevention of T2D, i.e. preventing IFG and IGT, could improve the population’s HRQoL, as the HRQoL has already diminished once a person has developed IFG or IGT. The early initiation of healthy lifestyle interventions is encouraged. Our results could motivate individuals with pre-diabetes to participate in prevention initiatives, mainly lifestyle modification. Further, policy makers and health care providers could consider to screen for pre-diabetes and to support programs to prevent T2D among those with pre-diabetes.

We encourage further evaluations of HUW instruments for the Swedish population. While we decided to only consider a small number of predictors for HUWs, estimations of the influence of other risk factors on HUWs are needed.

This analysis is part of health economics research to investigate the cost-effectiveness of diabetes prevention initiatives. Previous analyses revealed that data was missing for this broad aim [9], with information on HUWs lacking for people with T2D, pre-diabetes and NGT from one source population. Our research adds important information for a T2D prevention cost-effectiveness model using cost per QALY gained as measurement.

## Conclusions

This is the first study that estimated the HUW for NGT, IFG, IGT, IFG&IGT and T2D within the Swedish population. We found that, depending upon defined risk factors, the worse the glucose group, the lower the HUWs. We found that pre-diabetic states decrease the HRQoL compared to healthy individuals. This shows that, additionally to lowering the risk of developing T2D, preventing pre-diabetes would also improve the HRQoL. HUWs can be compared across states that define the natural history of T2D and allow the establishment of a T2D prevention model.

## Abbreviations

BMI: Body mass index
HRQoL: Health-related quality of life
HUW: Health utility weight
IFG: Impaired fasting glucose
IGT: Impaired glucose tolerance
NGT: Normal glucose tolerance
QALY: Quality adjusted life year
T2D: Type 2 diabetes mellitus
VIP: Västerbotten Intervention Program
